# A framework for an organelle-based mathematical modeling of hyphae

**DOI:** 10.1186/s40694-015-0014-2

**Published:** 2015-07-21

**Authors:** Rudibert King

**Affiliations:** Chair of Measurement and Control, Technische Universität Berlin, Berlin, Germany

**Keywords:** Morphological model, Hypha, Vesicles

## Abstract

**Background:**

Although highly desirable, a mechanistic explanation for the outstanding protein secretion capabilities of fungi such as *Aspergilli* is missing. As a result, a rational and predictive design of strains as cell factories for protein production is still out of reach. The analysis of the secretion apparatus is not only hampered by open issues concerning molecular cell biological processes, but as well by their spatial fragmentation and highly dynamic features. Whereas the former issues are addressed by many groups, an account of the space- and time-dependent processes, which is best done by means of mathematical models, is lacking. Up to now, mathematical models for hyphal organisms mainly focus on one of two extremes. Either macroscopic morphology, such as pellet or mycelium growth, is addressed, or a microscopic picture is drawn predicting, for instance, the form of a hyphal tip. How intra-hyphal transport and organelle distribution works, however, has not been tackled so far mathematically.

**Results:**

The main result of this contribution is a generic modeling framework to describe the space- and time-dependent evolution of intracellular substances and organelles. It takes intrahyphal, passive and active transport of substances into account and explains exponential and then linear length growth by tugor-driven uptake of water. Experimentally observed increasing concentration levels of organelles towards the tip can be well explained within the framework without resorting to complex biological regulations. It is shown that the accumulation can be partly explained by geometrical constraints, besides a necessary deceleration of the active transport velocity. The model is formulated such that more intricate intracellular processes can be included.

**Conclusions:**

Results from steady-state experiments are easy to be interpreted. In a hyphal network, however, new branches are produced at an exponential rate. Moreover, passive and active transport processes give rise to a spatial distribution of organelles and other cytoplasmatic constituents inside hyphae. As a result, most of the data obtained in experiments will be from a non-steady and space dependent state. A quantitative and mechanistic explanation of the processes occurring will only be possible if these dependencies are taking into account while evaluating experimental findings.

## Background

The ecological and technical relevance of fungi is outstanding. They are integrated in most ecosystems, act as detrimental agents for plants and humans, decompose waste materials, and are exploited in the synthesis of valuable products [1, 2], to name just a few areas in which they play a major role. Their most striking feature is polarized growth and branching which leads to more or less dense mycelia or pellets [3, 4]. Concomitantly, *Aspergilli* such as *A. niger*, *A. oryzae* and *A. terreus* have astounding capabilities to secrete interesting enzymes, mainly through the apical region [5]. A rational design to obtain modified strains as optimized cell factories, however, is still limited by the incomplete picture of their growth, production and secretion machinery. By the very nature of living cells all occurring processes are highly dynamic and the behavior of a cell does not only depend on the actual stimuli but what had happened to the cell in the past. For fungal organisms, interpretation of physiological data is even more challenging. Besides the compartmentalization of biological functions in distinct organelles, space- and time-dependent distributions occur. This relates to organelles, other cytoplasmic compounds, and stimuli in and around a mycelium, and, therefore, impede the deduction of meaningful knowledge and hypotheses. All of which could be addressed in the context of mathematical models.

**Figure 1:**
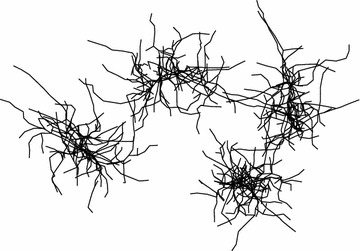
Simulated 3D-growth of mycelia projected to two dimensions.

So far most mathematical models for hyphal organisms respecting morphological features focused on macroscopic processes in dense mycelia and pellets. Here, the interplay between nutrient transport by diffusion and space-dependent growth was addressed, see for example [1, 6–18] and references therein. A second group of models tries to predict the geometric appearance of rather small mycelia [19–25]. In these models, very little detailed biological information is needed, if used at all, to give rather realistic pictures. The results of simulations shown in Figure 1, as an example, just use the information that the apical growth rate in three dimensions is constant and that septa and branches are formed when a critical length of a hyphal compartment is obtained. Including a random growth direction in the simulations gives a visual impression which is hard to distinguish from real photographs. As for the first group of models, these models will not help in deciphering the secretion apparatus. This is true as well for so-called morphologically structured models, which do not even account for the space-dependency, see, e.g., [7, 21, 26].

A more detailed account of microscopic features of individual hyphae is given by a last group of models to describe, for instance, the shape of a tip, or the growth in length. A well-known example for the first class of problems, which will be used in what follows, is given by Bartnicki-Garcia et al. in [27]. Here, the geometrical form of an apex is predicted with a simple model. It is based on a set of hypotheses how vesicles are transported ballistically from the Spitzenkörper to the wall. The model has been refined in future works to better account for the three-dimensional shape of a tip, the way vesicles are transported to the wall by diffusion, by representing the cell wall as a flexible membrane, or by a better account of vesicle fusion with the cell wall [28–32].

Mathematical descriptions of the growth in length, as the second class of microscopic features, were given recently with two different approaches. In [33], the long-range transport of material in hyphae is depicted by a particle transport along a single, hypothetical microtubule extending over the whole length of a hypha. The amount of material reaching the tip of the hypha determines length growth. A changing velocity, however, is neither considered nor a movement of the microtubules with the cytoplasmatic flow. In contrast, [34] explain length growth of *Phanerochaete velutina* mathematically by a turgor driven intra-hyphal flow towards the tip. In all these approaches a constant length growth rate is considered which is not true for the germ tube. Moreover, new branches of a mycelium very often show a lower initial velocity as well. As a result, and as a mycelium grows exponentially by an exponential production of new branches, a significant part of a mycelium will not be in a kind of quasi-steady state which is assumed above. In major parts of a mycelium, organelles and intracellular substances have not yet reached their quasi-steady state distribution, which might be important for a quantitative prediction of the growth of the mycelium. Likewise, if septa are closed and opened by Woronin bodies, intra-hyphal flow has to stop or will resume resulting in even more complex situations.

In our former works [11, 19], we explained the initially observed exponential and then linear growth with the limitation by a hypothetical intracellular compound. We had to resort to a hypothetical compound at that time as neither for fungi nor for actinomycetes details about the mechanism were known. Especially for fungi, this situation has changed drastically in recent years. Molecular methods, bioinformatics and image analysis have provided us with a whelm of information if not give rise to a ‘Big Data Tsunami’ [35]. More specifically, for the processes addressed here, which are responsible for length growth and (product) secretion, much more is known today. Excellent recent reviews about growth in length of fungal hyphae are given, e.g., by [36, 37], and about secretion in [32]. The importance of turgor driven length extension is stretched by [38, 39] in a series of papers.

We therefore think that the time is ripe to try to condense at least a small part of the available knowledge in a mathematical model. This can form the basis to discuss hypotheses and to account for the effects of space- and time-dependencies in the interpretation of experimental data. The model structure derived in this contribution therefore serves two main purposes.

- First of all, it represents a basic model structure, with which the initial exponential and then linear growth of a hypha can be described with a minimal amount of assumptions. This turgor driven evolution of the intra-hyphal flow forms the ‘backbone’ for all other processes occurring in a hyphae, and, therefore, has to be considered first. To be more specific, besides the postulation of some kinetics, no further biological regulations will be introduced to describe the experimentally observed growth evolution. If this is possible, already simple physical transport processes combined with implicitly formulated regulations through kinetic expressions can be used to explain the observed behavior without resorting to complex biological mechanisms. This, of course, does not rule out such regulations which additionally may occur. If experimental evidence is given, these processes can be included readily.
- Secondly, the model structure derived serves as a basis for future work when experimental data is interpreted and condensed in a mathematical framework. As an example, the distribution of vesicles in a hypha will be considered here which shows a distinct profile along the length of a hypha. Again simple physical arguments, mainly with respect to the active transport velocity and the geometry of the tip, will be enough to explain experimental data where a significant increase in concentration is observed toward the tip.

In the long run, such kind of models might help in answering questions raised in the endeavor toward a rational strain design. Examples are [5]: How many vesicles carrying proteins of interest can be used without interfering with vesicles for growth? Where are the bottlenecks in vesicle-mediated protein secretion? How many proteins can be channeled through the secretory pathway in order to provide each protein sufficient time to become correctly folded? Extending this list of questions will naturally occur when a model is at hand.

The rest of the paper is organized as follows. After a problem statement in the next section, the model of Bartnicki-Garcia et al. is revisited to determine the volume and surface area in the tip region. This will then be used to correct experimental data. The general model is formulated next. As a first application, length growth by turgor driven water uptake is described. Extending the model with vesicles allows for a comparison with experimental data in the last section before the paper finishes with some conclusions.

## Problem statement

What will be described below, will neither encompass the process of sporulation nor branching. An attempt to model germination can be found, e.g., in [40]. It is assumed that a very short part of a tip already exists, presumably from a mother hypha. In the simulations shown, depending on the boundary conditions, the mother hypha will either have no influence on the developing branch or it will supply material for growth. After some time, say $t=t_t$, the retrograd end of the new hypha will posses a final radius of $r_t(t_t)=R$ and the length of the tip will be $L_t(t_t)=L_{tmax}$. For $t>t_t$, the hypha will consist of this constant tip part with fixed length $L_{tmax}$ and a seemingly growing distal region of length $L_d(t)$, i.e., the overall length of the hypha will then be given by

1 $$\begin{aligned} L(t) = L_d(t) + L_{tmax}. \end{aligned}$$

Consequently, it will be assumed that, after tip completion, its geometrical form and size stay constant. Both assumptions together simplify the mathematical treatment significantly as apparent growth is associated to a subapical region where the cell wall becomes rigidified, probably by the action of cross-linking enzymes [37]. With this assumption, the complex processes involved in plasma membrane and cell wall synthesis actually occurring in the tip region are approximated. A more detailed model is given by [32]. A constant form and size of the tip region, on the other side, necessitates a constant radius of the distal part. This is in contrast to the model introduced in [34] where the authors correlate high internal volume flows *Q* with large diameters of hyphae. This will not be considered here for simplicity although the general model could account for it.

**Figure 2:**
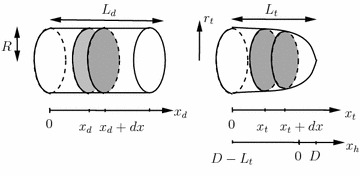
Sketch of the distal part (*d*) with a constant radius *R*, and the tip (*t*) of a hypha.

For a simpler numerical implementation, the tip and the distal part will be modeled separately with appropriate conditions to account for the connection between them, see Figure 2. In what follows, the variables related to the tip will be denoted by an index *t* whereas those related to the distal part with a *d*.

The general model will be obtained by a formulation of balance equations applied to an infinitesimal intra-hyphal balancing volume of length *dx*, see the space between shaded areas in Figure 2. Terms of production, consumption and transport via the cell membrane, the consequences of an intra-hyphal flow, and, finally, due to active translocation will be considered. Space-dependent uptake of nutrients could be included readily, but is not done here. Most importantly, a constant physiological and functional state is assumed along a hypha. If this is not the case, the model developed in this contribution has to be combined with approaches proposed, e.g., by Nielsen and Villadsen [18].  As the transport from and to the environment will be proportional to the local surface area and production and consumption rates will be given based on the local volume, these quantities have be determined first.

## Geometrical model of the tip

For simplicity, the 2D-model proposed in [27] describing the form of a hyphal tip is used to derive expressions for the local surface area $A(x_t)$ and volume $V(x_t)$. Although the initial conjecture that the actual 3D-form of a hypha can be produced by a rotation of the solution of the 2D-model was corrected in [28], see as well [29], the simpler approach is used here. This is motivated by the fact that the actual differences in the forms obtained are small while the calculation of the 3D-form is rather involved.

In [27] it is proposed that: (1) the cell surface expands from materials discharged by wall-destined vesicles, (2) vesicles are released from a postulated vesicle supply center (VSC), (3) vesicles move from the VSC to the surface in any random direction. Based on these propositions, they derive the following model.

If the VSC is located in the origin of an $(x_h, y_h)$-coordinate system, as used by Bartnicki-Garcia et al. [27], where the cartesian coordinate $y_h$ equals the radius $r_t$ in Figure 2, i.e., $y_h=r_t$, the 2D-geometry reads

2 $$\begin{aligned} x_h = r_t \cot \frac{\displaystyle r_t}{\displaystyle D}. \end{aligned}$$

In this coordinate system, the hypha extends to negative $x_h$-values. The foremost point of the tip is at $x_h=D$. With $x_t=x_h+L_t-D$, see Figure 2, the geometry of the tip is given by

3 $$\begin{aligned} x_t = r_t \cot \frac{\displaystyle r_t}{\displaystyle D} +L_t-D \end{aligned}$$

in the coordinates used in this work.

For the calculation of the volume $V(x_t)$ and surface area $A(x_t)$, $r_t(x_t)$ is locally approximated by a straight line of length $l_t$ connecting $r_t(x_t)$ and $r_t(x_t+dx)$. Rotating this line defines the area and the enclosed volume.

Neglecting higher order terms, the infinitesimal surface area of such a truncated cone is given by

4 $$\begin{aligned} A(x_t) \approx 2 \pi \sqrt{1 + \Big (\frac{dr_t}{dx_t}\Big )^2 } r_t(x_t) dx, \end{aligned}$$

and the infinitesimal balancing volume is approximated by

5 $$\begin{aligned} V(x_t) \approx \pi r_t^2(x_t) dx. \end{aligned}$$

The derivative needed in the former expression can be obtained from Eq. 3 by implicit differentiation, see Appendix A.

**Figure 3:**
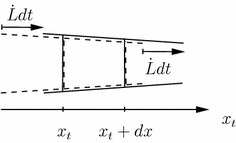
The balance volume for time instant *t* is depicted by a *box* with *broken lines* and for $t+dt$ with *solid lines*. As the tip moves to the *right*, the volume increases for a stationary observer.

For a stationary observer, see Figure 3, the balance volume increases when the tip grows out of a considered section $[x_t, x_t+dx]$.

As it is assumed that the form of the tip stays constant, every point of the surface moves with the extension rate $\dot{L}$ of the hypha, hence,

6 $$\begin{aligned} \dot{V}_t = 2 \pi r_t \frac{\displaystyle d r_t}{\displaystyle d x_t} \frac{\displaystyle d x_t}{\displaystyle dt} dx = 2 \pi r_t \frac{\displaystyle d r_t}{\displaystyle d x_t} (-{\dot{L}}) dx. \end{aligned}$$

A negative sign is included as for a stationary observer the volume increases while $d r_t/d x_t$ is negative.

The tip and the distal part must be connected without a step in radius, i.e., $r_t(x_t=0) = R$. As a result, *D* cannot be chosen independently when $L_{tmax}$ is fixed. From a biological point of view, fixing *D* might be a better alternative. This, however, would lead to a very long tip region which is ruled out here for numerical reasons. From Eq. 3, when $L_{tmax}$ is prescribed, an implicit expression is given for *D*

7 $$\begin{aligned} 0 = R \cot \frac{\displaystyle R}{\displaystyle D} +L_{tmax}-D. \end{aligned}$$

**Figure 4:**
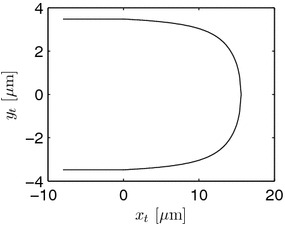
Cross-section of a simulated tip of a hypha for $x_t \ge 0$. Some distal part with constant radius is shown as well for $x_t<0$.

For small $L_{tmax}$, this value of *D* is an approximation of the real distance of the VSC from the apex. Figure 4 gives an impression of the obtained form of the tip, where some distal part is shown as well. The calculations were done with $R=3.5$ μm, $D=1.2$ μm, and $L_{tmax}=15.6$ μm.

## Experimental data

Although the main goal of this contribution lays in the derivation of a generic model structure, some comparisons with experimental data will be done.

**Figure 5:**
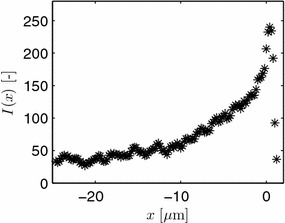
Relative expression intensity *I* of the fluorescent protein SynA::eGFP in *A. niger* as a function of the distance to the apex. Original unpublished data from F. Spanhoff et al.

**Figure 6:**
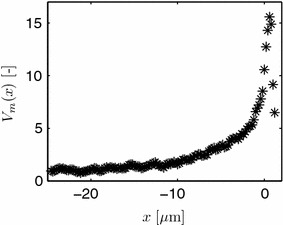
Corrected and normalized vesicle concentration $V_m$.

Unpublished experimental data with vesicle measurements from *Aspergillus niger* is kindly supplied by F. Spanhoff, A. Ram and V. Meyer. They visualized the secretory vesicle concentration of individual hyphae by the intensity of the fluorescent R-SNARE protein SynA using a Zeiss confocal microscope. Pictures were taken at an equidistant spacing of 0.2 μm along the length of a hypha for typically 10–12 layers (z-stacks) across the diameter. Hyphae were obtained from the periphery of a fungal colony. Uncalibrated fluorescence data is obtained by adding up all intensity values for each z-stack to obtain *I*. The mean value for 7 different hyphae is shown in Figure 5 as a function of the distance to the apex. The origin of the *x*-coordinate system used here is chosen $D=1.2$ μm behind the apex, which coincides with the VSC used later. As the scanned volume in the apical dome is smaller than in subapical regions, the data is corrected here for this geometrical effect. Again the model of [27] is used to calculate a local volume, and, hence, from the intensity data, a local normalized measured concentration $V_m$. As absolute information about the number of vesicles is missing, the data is additionally normalized by an arbitrary scaling factor of 35 to compare against simulation results later. The scaling factor is chosen such that in the subapical region a normalized concentration of approximately 1 is obtained. The corrected data is shown in Figure 6. Observe the higher ratio of maximal to subapical values in the corrected data compared to the given intensity values in Figure 5.

In the experiments described above, hyphal length was not measured for *A. niger* as a function of time for newly developing branches. Typically, an initial exponential growth will be observed followed by a linear one. As the model will be able to describe this, another set of data is used here for comparison. Experimental results from [41] are exploited. This rather old set of data was already used by us in [11]. In this former work, a much simpler model was proposed to describe length evolution. Using the data again, both approaches can be compared. Fiddy and Trince [41] measured the evolution of a primary branch of *Geotrichum candidum* extending out of an intercalary compartment just behind a septum. They observed a correlation of the decreasing length extension rate of this branch with septation occurring after some time in this branch. However, the extension rate of the primary branch continued to increase, despite septation, until a length of about 700 μm was reached. From Figure 3b in [41], a maximal extension rate of 2.5 μm/min can be estimated. The data will be given later together with the results of a simulation.

## Generic model

To derive a generic model, a substance $\mathcal{S}_i$, $i=d,t$ is balanced in a segment extending from $x_i$ to $x_i+dx$, see Figure 2. In what follows, $\mathcal{S}_t$ represents vesicles ($\mathcal{V}_t$), Spitzenkörper ($\mathcal{K}_t)$, osmolytes ($\mathcal{O}_t$), etc. in the tip, i.e., $\mathcal{S}_t = \{\mathcal{V}_t, \mathcal{K}_t, \mathcal{O}_t, \ldots \}$. Accordingly, $\mathcal{S}_d = \{\mathcal{V}_d, \mathcal{K}_d, \mathcal{O}_d, \ldots \}$ will denote variables in the distal part. A radial distribution inside the hypha and diffusion in all directions are neglected. Diffusion in the *x*-direction could be included readily without complicating the numerical solution much. It would, however, make less sense for organelles. See [39] for a discussion of diffusion coefficients of different cellular components compared against the intra-hyphal flow velocity.

The volumetric mass concentration of a substance $\mathcal{S}_i$ is represented here by the very same symbol in the equations, i.e., $\mathcal{S}_i$ is used to denote the concentration of the generic compound. Hence,

8 $$\begin{aligned} \mathcal{S}_i(x_i,t) = \frac{\displaystyle m_{\mathcal{S}_i}(x_i,t)}{\displaystyle V(x_i)} . \end{aligned}$$

Balancing the mass $m_{\mathcal{S}_i}$ of substance $\mathcal{S}_i$ in a segment of volume $V(x_i)$ with surface area $A(x_i)$ and infinitesimal length *dx* leads to

9 $$\begin{aligned} \frac{\displaystyle \partial m_{\mathcal{S}_i}}{\displaystyle \partial t} &= \Big ( \mu _{\mathcal{S}_ip} - \mu _{\mathcal{S}_ic} \Big ) V(x_i) + \mu _{\mathcal{S}_it} A(x_i) \nonumber \\ &\quad + Q_i(x_i,t) \mathcal{S}_i(x_i,t) - Q_i(x_i+dx,t) \mathcal{S}_i(x_i+dx,t) \end{aligned}$$

with production (${}_{\ldots p}$) and consumption (${}_{\ldots c}$) rates $\mu _{\mathcal{S}_ip}$ and $\mu _{\mathcal{S}_ic}$, respectively. Transport (${}_{\ldots t}$) from or to the surroundings is modeled by $\mu _{\mathcal{S}_it}$. Whereas the former reaction rates are given as a temporal change of mass per volume $V_i=V(x_i)$, the latter is based on the local external surface area $A_i=A(x_i)$ of the segment.

The last two terms in Eq. 9 represent intra-hyphal flow in and out of the balance volume, i.e., flow through the shaded areas in Figure 2. As due to turgor pressure hyphae take up water from the surroundings, and as only the apical region can extend in the real hypha, an intra-hyphal flow is set up. Hence, the volumetric flow rate $Q_i(x_i,t)$ is both a function of space $x_i$ and time $t$.

Intra-hyphal flow of a substance can be the result of the flow of the cytoplasm (*cyt*) transporting $\mathcal{S}_i$. For other substances, long-distance transport is realized via an active (*act*) dislocation along microtubules. In the latter case, microtubules can be transported as well with the flowing cytoplasma, see [42, 43], resulting in a superposition of flow and dislocation velocities for $\mathcal{S}_i$,

10 $$\begin{aligned} Q_i(x_i,t) = Q_{i,cyt}(x_i,t) + Q_{i,act}(x_i,t). \end{aligned}$$

Alternatively, it can be assumed that microtubules stay fixed with respect to the cell wall and that all of $\mathcal{S}_i$ is attached to them. Then, $Q_{i,cyt}=0$ for this specific substance.

The splitting up of $Q_i$ will now be used for a volume balance which is only affected by the cytoplasmic flow. As the cytoplasm is incompressible,

11 $$\begin{aligned} \frac{\displaystyle \partial V_i}{\displaystyle \partial t} = {\dot{V}_i} = Q_{i,cyt}(x_i,t) - Q_{i,cyt}(x_i+dx,t) + \mu _{V_ip} A(x_i) \end{aligned}$$

where $\mu _{V_ip}$ represents the volume production, e.g., through turgor-driven uptake of water from the environment through the local surface $A_i=A(x_i)$.

To finally set up the generic model structure based on the balances given above, several steps are necessary which are detailed in the Appendix B:

1. The last term of Eq. 9 and the second term of the right-hand side of Eq. 11 are expanded in a Taylor series, neglecting all terms in $(dx)^n$, $n \ge 2$.
2. All equations are combined.

This leads to

12 $$\begin{aligned} \frac{\displaystyle \partial \mathcal{S}_i}{\displaystyle \partial t} &= \mu _{\mathcal{S}_ip} - \mu _{\mathcal{S}_ic} + \rho _{i1} \mu _{\mathcal{S}_it} - \rho _{i1} \mu _{V_ip} \mathcal{S}_i \nonumber \\ &\quad - \rho _{i2} \mathcal{S}_i \frac{\displaystyle \partial Q_{i,act}}{\displaystyle \partial x_i} - \rho _{i2} Q_i \frac{\displaystyle \partial \mathcal{S}_i}{\displaystyle \partial x_i} \end{aligned}$$

with

13 $$\begin{aligned} \rho _{i1} = \frac{\displaystyle A_i}{\displaystyle V_i}, \quad \rho _{i2} = \frac{\displaystyle dx}{\displaystyle V_i}. \end{aligned}$$

Initial and boundary conditions, and $Q_i$ will be specified below.

### Generic model of the constant, distal part

For simplicity, it is assumed that the active translocation velocity along microtubules in the distal part, if it exists at all, is constant, i.e., for its gradient

$$\begin{aligned} \frac{\displaystyle \partial Q_{d,act}}{\displaystyle \partial x_d} = 0. \end{aligned}$$

Only at the tip, a deceleration will be considered later. Furthermore, after setting $i=d$, and $A_d= 2 \pi R dx$, $V_d= \pi R^2 dx$, Eq. 12 reads

14 $$\begin{aligned} \frac{\displaystyle \partial \mathcal{S}_d}{\displaystyle \partial t} = \mu _{\mathcal{S}_dp} - \mu _{\mathcal{S}_dc} + \rho _{d1} \mu _{\mathcal{S}_dt} - \rho _{d1} \mu _{V_dp} \mathcal{S}_d - \rho _{d2} Q_d \frac{\displaystyle \partial \mathcal{S}_d}{\displaystyle \partial x_d} \end{aligned}$$

with

15 $$\begin{aligned} \rho _{d1} = \frac{\displaystyle 2}{\displaystyle R}, \quad \rho _{d2} = \frac{\displaystyle 1}{\displaystyle \pi R^2}. \end{aligned}$$

The fourth term on the right hand side can be interpreted as a dilution term due to intra-hyphal flow. From Eq. 31, see Appendix, with $\dot{V}_d=0$, an expression can be given describing the spatial evolution of the intra-hyphal flow

16 $$\begin{aligned} \frac{\displaystyle \partial Q_{d,cyt}}{\displaystyle \partial x_d} = 2 \pi R \mu _{V_dp}. \end{aligned}$$

As long as the volume production $\mu _{V_dp} \ne 0$, Eq. 16 gives rise to a monotone increase of $Q_d$ with $x_d$, i.e., more and more fluid will be transported toward the tip.

The boundary conditions, $Q_d(0,t) = Q_{dx0}(t)$ and $\mathcal{S}_d(0,t) = \mathcal{S}_{dx0}(t)$, describe the information coming from a spore or from a branching site of a mother hypha. Whereas a mathematically ‘convenient’ boundary condition $Q_{dx0}(t)=$ const. would make sense, as it describes an active transport, which can be zero as well, $\mathcal{S}_d(0,t) = \mathcal{S}_{dx0}(t)=$ const. would be more difficult to justify biologically. This would mean that the mother hypha or spore would not change its value of $\mathcal{S}$ irrespective of what is going on in the new hypha. This could only be explained by a source of $\mathcal{S}$ of infinite strength. If the spore or the mother hyphae are not determined by a separate model,

$$\begin{aligned} \left. \frac{\displaystyle \partial \mathcal{S}_d(x_d,t)}{\displaystyle \partial x_d} \right| _{x_d=0} = 0 \end{aligned}$$

is a better choice, at least from a numerical point of view. An initial condition for $\mathcal{S}_d$ must not be specified as the simulation will start without a distal region.

Before specifying the individual production and consumption rates this generic model equation will be adapted to the non-constant-area and non-constant-volume case seen in the tip.

### Generic model of the tip

Due to the non-constant surface area $A_t$ and volume $V_t$ of the balancing volume the expressions get more involved. With Eqs. 4, 6 and 31 the resulting cytoplasm flow reads

17 $$\begin{aligned} \frac{\displaystyle \partial Q_{t,cyt}}{\displaystyle \partial x_t} = 2 \pi \sqrt{1 + \Big (\frac{dr_t}{dx_t}\Big )^2 } r_t(x_t) \mu _{V_tp} + 2 \pi r_t \frac{\displaystyle d r_t}{\displaystyle d x_t}{\dot{L}}. \end{aligned}$$

The first term on the right hand side increases the flow due to turgor driven volume production $\mu _{V_tp}$. The second term, however, as $dr_t/dx_t$ is negative, decreases the flow to account for the volume needed for length growth in the tip region.

**Figure 7:**
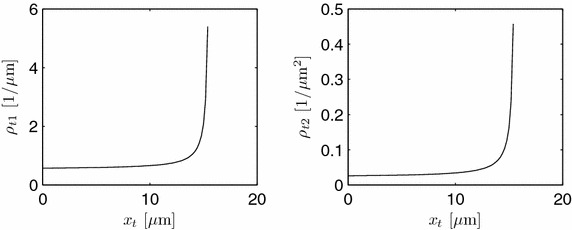
$\rho _i$’s for $R=3.5$ μm, $D=1.2$ μm, and $L_t=15.6$ μm.

Using Eq. 12,

18 $$\begin{aligned} \frac{\displaystyle \partial \mathcal{S}_t}{\displaystyle \partial t} &= \mu _{\mathcal{S}_tp} - \mu _{\mathcal{S}_tc} + \rho _{t1} \mu _{\mathcal{S}_tt} - \rho _{t1} \mu _{V_tp} \mathcal{S}_t \nonumber \\ &\quad - \rho _{t2} \mathcal{S}_t \frac{\displaystyle \partial Q_{t,act}}{\displaystyle \partial x_t} - \rho _{t2} Q_t \frac{\displaystyle \partial \mathcal{S}_t}{\displaystyle \partial x_t} \end{aligned}$$

with

19 $$\begin{aligned} \rho _{t1}(x_t) = \frac{\displaystyle 2\sqrt{1 + \Big (\frac{dr_t}{dx_t}\Big )^2 } }{\displaystyle r_t(x_t)}, \quad \rho _{t2}(x_t) = \frac{\displaystyle 1}{\displaystyle \pi r_t^2(x_t)} \end{aligned}$$

results. A plot of these $\rho _t$-terms is given in Figure 7. In the distal part, the corresponding terms are constant, see Eq. 15.

Besides an initial condition $\mathcal{S}_t(x_t,0) = \mathcal{S}_{tt0}(x_t)$, a boundary condition has to be specified. If a distal part is not yet formed,

$$\begin{aligned} \left. \frac{\displaystyle \partial \mathcal{S}_t}{\displaystyle \partial x_t}\right| _{x_t=0} = 0 \end{aligned}$$

is applied. If, on the other side, a distal compartment already exists,

20 $$\begin{aligned} \left. \mathcal{S}_t\right| _{x_t=0} = \left. \mathcal{S}_d \right| _{x_d=L_d}. \end{aligned}$$

### Model of length growth

As the radius of the distal part and the form of the tip stay constant, $\dot{L}$ is given by the overall volume produced divided by the area of the growing end. If the tip is still developing, this area is $\pi r_t^2$. Hence,

21 $$\begin{aligned} \frac{\displaystyle dL(t)}{\displaystyle dt} = \frac{\displaystyle 1}{\displaystyle \pi r_t^2} Q_{tp}(L_t,t). \end{aligned}$$

When the tip is finished, with $L_t=L_{tmax}$, it is assumed that length growth is realized by extension of the right hand side of the distal part with area $\pi R^2$, see Figure 2. For a tip with constant geometry, this translates into a growth in length of the hypha of

22 $$\begin{aligned} \frac{\displaystyle dL(t)}{\displaystyle dt} = \frac{\displaystyle 1}{\displaystyle \pi R^2} Q_{tp}(L_t,t). \end{aligned}$$

Here, a hypothetical flow rate $Q_{tp}(L_t,t)$ at the tip $x_t=L_t$ has to be used. It is determined by the gross increase of the volume flow rate in the tip region

23 $$\begin{aligned} \frac{\displaystyle \partial Q_{tp}(x_t,t)}{\displaystyle \partial x_t} = 2 \pi \sqrt{1 + \Big (\frac{dr_t}{dx_t}\Big )^2 } r_t(x_t) \mu _{V_tp}, \end{aligned}$$

with a proper boundary condition for $x_t=0$. Actually, the real flow rate in the tip region has to decrease towards the tip as it is ‘consumed’ everywhere in the tip to fill up the new volume formed due to tip growth. In the model, however, length increase is attributed to an increase in the distal part or left hand end of the tip before it is fully developed. Therefore, as volume is taken up in the tip region as well, the gross increase of the volume flow rate has to be known to determine $\dot{L}(t)$. To distinguish this hypothetical from the real flow rate, it is denoted by $Q_{tp},$ instead of $Q_{t,cyt}$. The boundary condition at the left hand side of the tip, see Figure 2, is $Q_{tp}(0,t) = Q_d(L_d,t)$ or 0 when only the tip exists.

### Complete generic model

In summary, after specifying the individual production, consumption and transport rates, $\mu _j$, and the change in active transport in the tip, $\partial Q_{t,act} / \partial x_t$, the following equations have to be solved in the *generic model* assuming that the tip has already reached its maximal extension:

1. Integration of Eq. 16 determines the intra-hyphal flow rate at $x_d=L_d$ which sets the boundary condition for Eq. 23.
2. Integration of latter equation leads to the hypothetical flow rate at the tip, $Q_{tp}(L_t,t)$,
3. and, with Eq. 22, to the actual extension rate $\dot{L}$.
4. In a moving boundary framework, as *L*(*t*) grows, Eqs. 14 and 18 are solved to determine $\mathcal{S}_i$, $i=d,t$.

Initially, only the tip region exists. Hence, Eqs. 16 and 18 are not needed.

## Modeling pressure regulation via osmolytes

We consider osmolytes which are responsible for maintaining a certain pressure and pressure gradient inside hyphae, see [38]. It is assumed that osmolytes are produced until a certain pressure is obtained for which intracellular sensors must exist. For the MAPK pathway, OS-1 is discussed as a sensor in [39]. For simplicity, an intracellular substance called osmolyte $\mathcal{O}_i$, $i=d, t$, is introduced, which represents both the osmolyte, and, indirectly, the pressure. To obtain a mass flow toward the apex, its concentration must be higher in subapical parts. Using the equations derived above, $\mathcal{S}_i$ is now replaced by $\mathcal{O}_i$.

To start with the most simple model, it is assumed that osmolytes are not transported actively, $Q_{i,act}=0$, and are not consumed or degraded, i.e.,

$$\begin{aligned} \mu _{\mathcal{O}_ic}=0. \end{aligned}$$

Furthermore, they are not transported over the cell wall, hence,

$$\begin{aligned} \mu _{\mathcal{O}_it}=0. \end{aligned}$$

For the production of $\mathcal{O}_i$, as a first approach, a logistic law-like expression is used

24 $$\begin{aligned} \mu _{\mathcal{O}_ip} = k_1 (\mathcal{O}_{max} - \mathcal{O}_i), \quad i=d,t, \end{aligned}$$

with a maximal osmolyte concentration $\mathcal{O}_{max}$. It has to be pointed out that these assumptions can be changed easily.

In the long run, for all osmolytes produced, water has to be taken up. If this process is fast, it can be assumed that, in a kind of quasi-steady-state point of view, water is taken up proportional to the synthesis rate of the osmolytes. As the first one is formulated based on the surface and the latter based on the volume,

$$\begin{aligned} \mu _{V_ip} A_i \sim \mu _{\mathcal{O}_ip} V_i \end{aligned}$$

leads to an expression for the volume production rate $\mu _{V_ip}$ in part *i* of the hypha

$$\begin{aligned} \mu _{V_ip} \sim \mu _{\mathcal{O}_ip} \frac{\displaystyle V_i}{\displaystyle A_i}. \end{aligned}$$

With Eq. 24, for the distal part,

25 $$\begin{aligned} \mu _{V_dp} = k_2 (\mathcal{O}_{max} - \mathcal{O}_d) \frac{\displaystyle V_d}{\displaystyle A_d} = k_2 (\mathcal{O}_{max} - \mathcal{O}_d) \frac{\displaystyle 1}{\displaystyle \rho _{d1}} \end{aligned}$$

and for the tip

26 $$\begin{aligned} \mu _{V_tp} = k_3 (\mathcal{O}_{max} - \mathcal{O}_t) \frac{\displaystyle V_t}{\displaystyle A_t} = k_3 (\mathcal{O}_{max} - \mathcal{O}_t) \frac{\displaystyle 1}{\displaystyle \rho _{t1}} \end{aligned}$$

follows. Different constants, $k_2$ and $k_3$, are introduced to possibly account for the fact that most water is taken up in the tip region, as hypothesized by [39]. This difference in water uptake velocity might be the result of the plasticity of the wall in the tip region. As the maximal extend of the tip, $L_{tmax}$, considered here is fixed arbitrarily, this has to be observed during parameter identification and analysis of the simulation results.

With these kinetics and the generic equations derived in the last section, the models of the distal part and the tip can be formulated. They are omitted here for brevity. Furthermore, to reduce the number of kinetic parameters and to ease parameter identification, a normalization is done with $o_i = \mathcal{O}_i / \mathcal{O}_{max}$ and $q_i = Q_{i,cyt} / Q_{max}$, and $o_i, q_i \in [0,1]$ for $i=d,t$, $\dot{L}_{max}=Q_{max}/(\pi R^2) = Q_{max} \rho _{d2}$, $\theta _1 = k_1$, $\theta _2 = k_2 \mathcal{O}_{max}$, $\theta _3 = k_3 \mathcal{O}_{max}$.

For the numerical solution of the partial differential equations, the spatial coordinate is discretized equidistantly with a step size of $\Delta x=0.2$ μm. The method of lines is applied for the equation describing the evolution of the osmolytes approximating the spatial derivatives by a first-order backward difference operator. In the beginning, when only the tip exists, the left most discretization segment of the tip, see Figure 2, is allowed to grow in length according to $\dot{L}$ until it exceeds a length of 0.3 μm. After the distal part is formed, its right most discretization segment takes over this task and grows accordingly until it exceeds a length of 0.3 μm. Then, this segment is split up into a segment of constant length (0.2 μm) and a growing one with an initial length <0.2 μm and the calculations are continued as before. The normalized flow rate $q_i$ is obtained accordingly from Eqs. 16 and 23 exploiting the trapezoidal rule. Parameters $\theta _i$ have to be chosen such that $q_i \le 1$ is guaranteed.

As this work concentrates on the formulation of the generic model and not on a parameter fit or selection of appropriate kinetic expressions to describe a very specific problem, a simple approach was chosen to find kinetic parameters for simulation studies. Measurements performed by Spanhoff et al. were done with hyphae growing with approximately ${\dot{L}}_{max} = 3 \mu$m/min. The parameters $\theta _{1,2,3}$ are determined such that *L*(*t*) shows an initial exponential increase followed, after a transition, by a phase of constant growth velocity of approximately ${\dot{L}}_{max} = 3 \mu$m/min. To this end, an optimization problem was formulated. In lack of real data for this first study, a ’desired’ evolution $L_{des}(t)$ was determined to allow for an adaption of the $\theta _i$’s.

**Figure 8:**
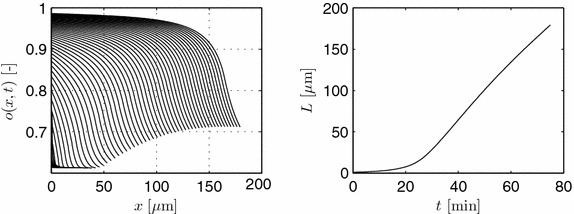
*Left* evolution of the normalized osmolyte concentration for different time instants with $\Delta t = 1$ min. *Right* length of the hypha.

**Figure 9:**
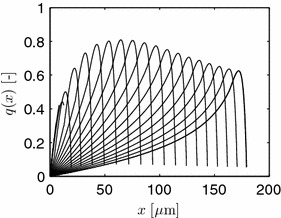
Normalized intrahyphal flow with $\Delta t = 3$ min. Parameters as in Figure 8.

Simulation results are shown in Figures 8 and 9. Parameters used are $\theta _1=0.3079$, $\theta _2=0.2619$, and $\theta _3=0.5032$ with vanishing flow from the mother hypha or spore. The initial tip starts with four discretization segments, i.e., a length of 0.8 μm, with exponentially decaying values of the initial, normalized osmolyte concentration $o_{t,k}= [0.750$, 0.7351, 0.7206, 0.7063].

In the simulations given in Figure 8, growth is followed up to a length of about 170 μm, i.e., in the end more than 850 discretization segments are used. Due to production and consumption of the osmolyte, and due to length growth, the osmolyte profiles change dynamically over time. These osmolyte profiles, on the other hand, determine the overall volume production, see Figure 9, and, hence, length increase. As a result, all processes are highly interwoven.

**Figure 10:**
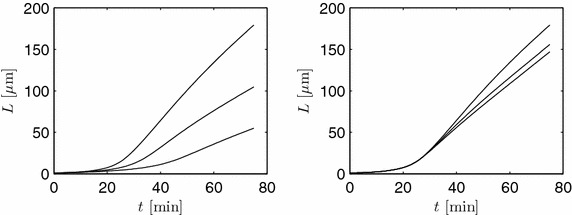
*Left* length evolution for $\theta _1$ = 0.3079(top)/0.3579/0.4079.* Right* Length evolution for $\theta _2$ = 0.2619(top)/0.2119/0.1619.

**Figure 11:**
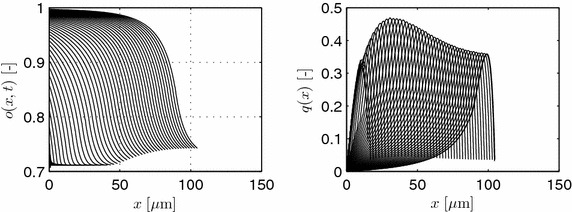
Normalized osmolyte concentration and flow rate for $\theta _1$ = 0.3579. The remaining parameters are as in Figure 8.

When the rate of osmolyte production is increased by choosing higher values of $k_1=\theta _1$, slower growth results, see left part of Figure 10. Here, as in the right part of this figure, the other parameters are as in Figure 8. Lowering $\theta _2=k_2 \mathcal{O}_{max}$ has the similar, though less pronounced effect. An explanation of this feature can be seen in Figure 11 where the evolutions of the osmolyte and the normalized flow rate are shown for an increased value of $\theta _1=0.3079 + 0.05$ compared to the case of Figure 8. A larger osmolyte production rate leads to higher values of *o*(*x*, *t*), and, hence, to a lower volume production rate $k_i (\mathcal{O}_{max} - \mathcal{O})$, $i=2,3$.

Using the same volume production parameters $\theta _2=\theta _3=0.2616$ in the distal and tip region results in a much too slow growth corroborating different uptake rates. These simple studies show that an intricate relation exists between parameters and the processes described by the model. Predicting the outcome of parameter or kinetic variations is difficult. Therefore, numerical studies are indispensable.

**Figure 12:**
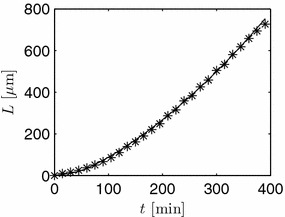
Length of a hypha. Simulation (*solid line*) done with $\theta _1=0.4792$, $\theta _2=0.4811$, $\theta _3=0.5282$, and $q_i(0,t)=0.0357$. Experimental data obtained by [41] are marked with *asterisk*.

In the first simulation study shown above, which will be used again in the next section, linear growth already starts at a length of approximately $L=30$ μm. By another choice of parameters, the exponential phase can be elongated. In Figure 12, as an example, parameters are chosen such that experimental data obtained by [41] can be described by the model. In this case, an elongated exponential phase can be observed. Truly linear growth does not start before 700 μm as pointed out by the authors. As the primary branch considered in [41] originates from a parent compartment, the normalized flow rate coming from this compartment was fitted as a parameter as well. For simplicity, a constant value $q_i(0,t)$ was assumed. A more detailed study should be performed, though, based on the general model introduced above, to better account for a variable influence of the mother compartment. Likewise, septation could be included. This would lead to a leveling out of the profiles inside the mother compartment and a variable supply of the new branch. Such a detailed study, however, is outside the scope of this contribution and has to be postponed to future work.

## Modeling of vesicle distribution

As a first example of an organelle, vesicles are considered which have been already used above to get an idea of the form of the tip. They are, beside other functions, responsible for material transport to the growing tip. According to the model of [27], vesicles are used up in the apical part of a hypha. Therefore, the consumption rate in the tip will be $\mu _{\mathcal{V}_tc} \ne 0$, while $\mu _{\mathcal{V}_dc} = 0$ is assumed in the distal region.

As vesicles are produced inside hyphae, the transfer rate to the surroundings is zero in both parts, i.e., $\mu _{\mathcal{V}_it}$, $i=d,t$. For vesicle production $\mu _{\mathcal{V}_ip}$, again a logistic-like expression is chosen. However, as vesicles are accumulated in the tip region, the expression is modified describing a production only as along as $\mathcal{V}_i < \mathcal{V}_{max}$ and no production otherwise

27 $$\begin{aligned} \mu _{\mathcal{V}_ip} = \text{ max } \Big \{k_4 ( \mathcal{V}_{max}- \mathcal{V}_i ), 0 \Big \}. \end{aligned}$$

Active transport is modeled by microtubules. It is assumed that all vesicles are bound immediately to microtubules. Therefore, the vesicle concentration can be calculated as the product of the local microtubules concentration, $m_{MT}/V$, times the loading of the microtubules, $m_{\mathcal{V}_i}/m_{MT}$

$$\begin{aligned} \mathcal{V}_i = \frac{\displaystyle m_{MT}}{\displaystyle V} \frac{\displaystyle m_{\mathcal{V}_i}}{\displaystyle m_{MT}} = \frac{\displaystyle m_{\mathcal{V}_i}}{\displaystyle V}. \end{aligned}$$

Due to the discrete nature of microtubules, this is a rough approximation. Still, if the local microtubules concentration stays constant up to the apex, the active volume flow is simply given by

$$\begin{aligned} Q_{i,act} = \pi r_i^2 u(x_i,t), \end{aligned}$$

where $u(x_i,t)$ and $r_i$ represent the local transport velocity and hyphal radius, respectively. With this expression, the volume flow at the apex is zero which does make sense as $r_t(L_{tmax})=0$. The change in flow rate over the length of a hypha is given by

$$\begin{aligned} \frac{\displaystyle \partial Q_{i,act}}{\displaystyle \partial x_i} = 2 \pi r_t u(x_i,t) \frac{\displaystyle d r_i}{\displaystyle d x_i} + \pi r_i^2 \frac{\displaystyle d u}{\displaystyle d x_i}. \end{aligned}$$

In the distal part, with $r_i=R =$ const.,

$$\begin{aligned} \frac{\displaystyle \partial Q_{d,act}}{\displaystyle \partial x_d} = \pi R^2 \frac{\displaystyle d u}{\displaystyle d x_d} = \frac{\displaystyle 1}{\displaystyle \rho _{d2}} \frac{\displaystyle d u}{\displaystyle d x_d}. \end{aligned}$$

For a constant velocity *u*,

$$\begin{aligned} \frac{\displaystyle \partial Q_{d,act}}{\displaystyle \partial x_d} = 0, \end{aligned}$$

as assumed above. For the tip,

$$\begin{aligned} \frac{\displaystyle \partial Q_{t,act}}{\displaystyle \partial x_t} = 2 \pi r_t u(x_t,t) \frac{\displaystyle d r_t}{\displaystyle d x_t} + \pi r_t^2 \frac{\displaystyle d u}{\displaystyle d x_t}. \end{aligned}$$

When the second term in the last equation is neglected, an increase in vesicle concentration, as seen in Figure 6, can only be observed directly behind the tip due to the decelerated transport coming from the geometrical influence ($dr_t/dx_t < 0$). This does not coincide with experimental findings. For this reason, as a first trial, it is assumed that *u* stays constant in most of the distal part at $u=u_{max}$. From $u_{max}$ it decreases linearly to zero in the foremost 15  μm. For the following simulation, $u_{max}=35.62$ μm/min is chosen. Typical literature values for kinesin-1 are in the range of 24–54 μm/min. For kinesin 7 in *A. nidulans*, [44] report 10 μm/min compared to the conventional kinesin of this strain with 120 μm/min.

The equations are normalized again with $v_i = \mathcal{V}_i /\mathcal{V}_{max}$ and $\theta _4 = k_4$.

Consumption of vesicles is assumed to be proportional to the actual length growth rate for which it is used, and proportional to the local vesicle concentration $v_t$

28 $$\begin{aligned} \mu _{\mathcal{V}_tc} = \theta _5 {\dot{L}} v_t. \end{aligned}$$

As the volume corrected measurements by Spanhoff et al. indicate a falling vesicle concentration only for the last three segments with a width of $\Delta x = 0.2$ μm, for the rest of the hypha $\mu _{\mathcal{V}_tc}=0$ is used.

**Figure 13:**
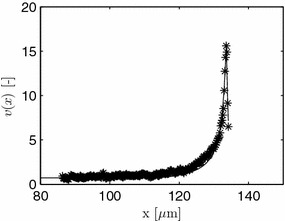
Comparison of a measured (*asterisk*) and simulated (*solid line*) evolution of normalized vesicle concentration as a function of the coordinate *x*, given for $t=60$ min. Only the foremost part of the hypha is given neglecting the retrograd part with constant *v*.

A comparison of simulated data against the measurements introduced above is given in Figure 13. Additional parameters used are $\theta _4=0.0407$, and $\theta _5=2.2701$. The remaining parameters $\theta _i$, $i=1,2,3$, are as in Figure 8.

With these parameters the experimentally observed accumulation profile of vesicles can be reproduced. A key-enabler is the model assumption that the transport velocity must decrease towards the tip. This could be tested in future experiments.

## Conclusions

As constantly new branches and septa are produced in a mycelium and a flow of cytoplasma towards the different apices occurs, quasi-steady state concentration profiles of intracellular substances and organelles hardly establish. Moreover, due to these processes and due to active and cytoplasmatic flows intracellular components are not only a function of time but as well a function of space inside a hypha. A comprehensive and quantitative interpretation of experimental data of individual hyphae will therefore only be possible when these space- and time-dependent processes are taken into account. To this end, a generic mathematical model is proposed here which first of all describes turgor driven length extension. By this, an initially accelerated and then linear growth can be predicted as seen in microscopic experiments. A much simpler model, see [11], could be used to predict the length evolution though. That model, however, cannot to be extended so easily to describe other constituents of a hypha as it is done here. The turgor driven length extensions forms the ‘backbone’ of a generic model to study, for instance, the time-dependent distribution of organelles and other compounds. These may be transported actively or passively towards the tip. Diffusion is not considered yet, but can be included readily. For a complete specification of the model, kinetic expressions have to be stated. In this contribution, very little effort is invested to study the effect of different kinetics and parameters, e.g., with respect to osmolyte or vesicle production and consumption. The emphasis is rather on the formulation of a generic model. Effects of different kinetics will be tackled in future works when more experimental data is available. However, even with simple kinetics chosen here it can be shown, for example, that the experimentally observed accumulation of vesicles near the tip can be explained. A crucial assumption to be able to do this is the postulation of a decreasing active transport velocity in the tip region. Without this, the experimentally observed apical accumulation of vesicles cannot be described in the chosen setting. The model structure can be readily extended to study the effect of different organelles and cytoplasmatic compounds. Before doing so, however, the numerical solution of the partial differential equations with a moving boundary should be revisited to hopefully decrease the computational burden. This was not done yet, as septation and branching have to be included in future works.

## Appendix A: Gradient of the radius

From Eq. 3,

$$\begin{aligned} F(x_t,r_t(x_t)) = r_t \cot \frac{\displaystyle r_t}{\displaystyle D} +L_t-D - x_t = 0. \end{aligned}$$

Implicit differentiation leads to

$$\begin{aligned} \frac{\displaystyle dr_t}{\displaystyle dx_t} = - \frac{\displaystyle \frac{\partial F}{\partial x_t}}{\displaystyle \frac{\partial F}{\partial r_t}} = \frac{\displaystyle D \sin ^2 \frac{\displaystyle r_t}{\displaystyle D}}{\displaystyle D \cos \frac{\displaystyle r_t}{\displaystyle D} \sin \frac{\displaystyle r_t}{\displaystyle D} - r_t }. \end{aligned}$$

## Appendix B: Derivation of the generic model

Expanding the last term in Eq. 9 into a Taylor series results in

$$\begin{aligned} Q_i(x_i+dx,t) \mathcal{S}_i(x_i+dx,t) &= Q_i(x,t) \mathcal{S}_i(x,t) \\ &\quad + \mathcal{S}_i \frac{\displaystyle \partial Q_i}{\displaystyle \partial x_i} dx + Q_i \frac{\displaystyle \partial \mathcal{S}_i}{\displaystyle \partial x_i} dx + \cdots \end{aligned}$$

This yields upon substitution and neglect of terms in $(dx)^n$, $n \ge 2$

$$\begin{aligned} \frac{\displaystyle \partial m_{\mathcal{S}_i}}{\displaystyle \partial t} = \Big ( \mu _{\mathcal{S}_ip} - \mu _{\mathcal{S}_ic} \Big ) V_i + \mu _{\mathcal{S}_it} A_i -\mathcal{S}_i \frac{\displaystyle \partial Q_i}{\displaystyle \partial x_i} dx - Q_i \frac{\displaystyle \partial \mathcal{S}_i}{\displaystyle \partial x_i} dx. \end{aligned}$$

In the distal part, the balancing volume $V(x_d)=V_d$ stays constant, i.e., $\dot{V}(x_d) = \dot{V}_d = 0$. In the tip region, however, a stationary observer sees a growing volume $\dot{V}(x_t) = \dot{V}_t$ as the apex moves away and the local radius increases. Hence, generally, with $m_{\mathcal{S}_i} = V_i \mathcal{S}_i$

$$\begin{aligned} V_i \frac{\displaystyle \partial \mathcal{S}_i}{\displaystyle \partial t} + \mathcal{S}_i \dot{V}_i &= \Big ( \mu _{\mathcal{S}_ip} - \mu _{\mathcal{S}_ic} \Big ) V_i + \mu _{\mathcal{S}_it} A_i \\ &\quad -\mathcal{S}_i \frac{\displaystyle \partial Q_i}{\displaystyle \partial x_i} dx - Q_i \frac{\displaystyle \partial \mathcal{S}_i}{\displaystyle \partial x_i} dx, \end{aligned}$$

or

29 $$\begin{aligned} \frac{\displaystyle \partial \mathcal{S}_i}{\displaystyle \partial t} &= \mu _{\mathcal{S}_ip} - \mu _{\mathcal{S}_ic} + \mu _{\mathcal{S}_it} \frac{\displaystyle A_i}{\displaystyle V_i} -\mathcal{S}_i \frac{\displaystyle \partial Q_i}{\displaystyle \partial x_i} \frac{\displaystyle dx}{\displaystyle V_i} - Q_i \frac{\displaystyle \partial \mathcal{S}_i}{\displaystyle \partial x_i} \frac{\displaystyle dx}{\displaystyle V_i} \nonumber \\ &\quad - \mathcal{S}_i \frac{\displaystyle \dot{V}_i}{\displaystyle V_i}. \end{aligned}$$

The surface area $A_i$ and volume $V_i$ will differ depending on the part considered.

Taylor series of the volume balance, see Eq. 11, which is only affected by the cytoplasmic flow, leads to

30 $$\begin{aligned} \frac{\displaystyle \partial V_i}{\displaystyle \partial t}&= {\dot{V}_i} \nonumber \\ &= Q_{i,cyt}(x_i,t) - Q_{i,cyt}(x_i+dx,t) + \mu _{V_ip} A(x_i) \nonumber \\ &\approx - \frac{\displaystyle \partial Q_{i,cyt}}{\displaystyle \partial x_i} dx + \mu _{V_ip} A_i. \end{aligned}$$

Rearrangement gives

31 $$\begin{aligned} \frac{\displaystyle \partial Q_{i,cyt}}{\displaystyle \partial x_i} dx = \mu _{V_ip} A_i - {\dot{V}_i}, \end{aligned}$$

and

$$\begin{aligned} \frac{\displaystyle \partial Q_{i}}{\displaystyle \partial x_i} dx &= \frac{\displaystyle \partial Q_{i,cyt}}{\displaystyle \partial x_i} dx + \frac{\displaystyle \partial Q_{i,act}}{\displaystyle \partial x_i} dx \\ &= \mu _{V_ip} A_i - {\dot{V}_i} + \frac{\displaystyle \partial Q_{i,act}}{\displaystyle \partial x_i} dx. \end{aligned}$$

Combining it with Eq. 29 leads to the *generic model*

32 $$\begin{aligned} \frac{\displaystyle \partial \mathcal{S}_i}{\displaystyle \partial t} &= \mu _{\mathcal{S}_ip} - \mu _{\mathcal{S}_ic} + \mu _{\mathcal{S}_it} \frac{\displaystyle A_i}{\displaystyle V_i} - \mu _{V_ip} \mathcal{S}_i \frac{\displaystyle A_i}{\displaystyle V_i} \nonumber \\ & \quad - \mathcal{S}_i \frac{\displaystyle \partial Q_{i,act}}{\displaystyle \partial x_i} \frac{\displaystyle dx}{\displaystyle V_i} - Q_i \frac{\displaystyle \partial \mathcal{S}_i}{\displaystyle \partial x_i} \frac{\displaystyle dx}{\displaystyle V_i}. \end{aligned}$$
